# Neuron-Specific Vitamin D Signaling Attenuates Microglia Activation and CNS Autoimmunity

**DOI:** 10.3389/fneur.2020.00019

**Published:** 2020-01-31

**Authors:** Priscilla W. Lee, Amanda Selhorst, Sara Gombash Lampe, Yue Liu, Yuhong Yang, Amy E. Lovett-Racke

**Affiliations:** ^1^Department of Microbial Infection and Immunity, The Ohio State University Wexner Medical Center, Columbus, OH, United States; ^2^Department of Neurology, The Ohio State University Wexner Medical Center, Columbus, OH, United States; ^3^Department of Neuroscience, The Ohio State University Wexner Medical Center, Columbus, OH, United States

**Keywords:** multiple sclerosis, experimental autoimmune encephalomyelitis, vitamin D, neurons, microglia

## Abstract

Low vitamin D during childhood is associated with an increased risk of developing multiple sclerosis (MS) as an adult. Given that vitamin D has anti-inflammatory properties, it has been postulated that the relationship between MS and low vitamin D is due to immune dysregulation. Since the vitamin D receptor (VDR) is expressed in many cell types, this study investigated an alternative hypothesis—neuron-specific VDR signaling induces anti-inflammatory molecules that protect the central nervous system from autoimmunity. Using media from neurons treated with calcitriol, the active form of vitamin D_3_, LPS-activated microglia had a reduction in pro-inflammatory molecules, and a reciprocal induction of anti-inflammatory molecules. Since IL-34 is critical to the homeostasis of microglia, and was previously shown to be induced in endothelial cells by vitamin D, we investigated IL-34 as the potential anti-inflammatory molecule induced in neurons by vitamin D. Treatment of LPS-activated microglia with IL-34 reduced pro-inflammatory cytokine production and enhanced the expression of anti-inflammatory transcripts. However, neutralizing IL-34 in vitamin D neuronal conditioned media only impacted IL-6 and not the broader anti-inflammatory phenotype of microglia. To mimic low vitamin D in children, we used a neuron-specific inducible mouse model in which VDR was partially deleted in juvenile mice. Partial deletion of VDR in neurons during early life resulted in exacerbated CNS autoimmunity in adult mice. Overall, the study illustrated that vitamin D signaling in neurons promotes an anti-inflammatory state in microglia, and low vitamin D in early life may enhance CNS autoimmunity.

## Introduction

Multiple Sclerosis (MS) is an inflammatory demyelinating disease of the central nervous system (CNS) that causes progressive neurological deficits, which affects over a million people in the USA. The geographical spread of MS has the lowest frequency along the equator, and increases in prevalence with increasing latitude (1–4). The risk of developing MS is largely determined before the age of 15 years (5–8) or at least within the first two decades (9), suggesting a role for the environment in modifying MS risks during childhood and adolescence. One of the strongest correlates of latitude is the duration and intensity of sunlight, and the synthesis of vitamin D is directly affected by ultraviolet B radiation. The incidence gradient according to latitude and the effect of migration within genetically uniform groups can be explained by vitamin D as the link between latitude and MS risks. The vitamin D hypothesis is supported by studies of sunlight exposure history. The seasonal fluctuations in vitamin D levels resulted in decreased vitamin D concentrations *in utero*, which contributed the month-of birth effect in MS (10). Higher sunlight exposure during childhood was shown to be associated with reduced MS risks (11–13). Vitamin D-rich diets and vitamin D supplementation have also been shown to reduce the risk of developing MS (14, 15). These epidemiology studies give credibility to the hypothesis that vitamin D, especially in early life, has a protective effect on MS development.

Vitamin D_3_ undergoes hydroxylation to generate 25-hydroxyvitamin D_3_ [25(OH)D_3_] in the liver, the main circulating form of the vitamin D with a relatively long half-life, and then converts to 1,25-dihydroxyvitamin D_3_ [1,25(OH)_2_D_3_, also known as calcitriol] in the kidney, the biologically active hormone (16). Calcitriol is the ligand for vitamin D receptor (VDR), a member of the nuclear receptor family of transcription factors which activates or represses the expression of nearly 1,000 genes in many cell types given the wide distribution of the vitamin D receptor [VDR; (17)].

After the onset of MS, vitamin D also acts in modulating MS clinical course. Serum concentrations of 25(OH)D_3_ in MS patients were lower during relapses than remissions (18), and correlated inversely with disease severity (19) and frequency of relapse (20, 21). Although these results might indicate lower sun exposure in patients with severe MS rather than a beneficial effect of vitamin D, convincing studies in the experimental autoimmune encephalomyelitis (EAE) model of MS have demonstrated the immunomodulatory effect of vitamin D in inflammatory CNS disease. Vitamin D suppresses EAE in prevention (22, 23) and therapeutic (24) studies. Moreover, the therapeutic effects of vitamin D required VDR function in T cells (25), and mediated by promoting IL-4, TGF-β (26) and IL-10 (27) production, and inhibiting T_H_1 cells differentiation (28, 29). Supplementation in juvenile animals was significantly more effective at reducing EAE susceptibility than adult supplementation (30), suggesting that vitamin D in early life can alter susceptibility to CNS autoimmunity.

Vitamin D has important effects on the development and function of the CNS. Neurons and microglia express VDR, and they can directly metabolize 25(OH)D_3_ because they express 1-α hydroxylase (31). 1,25(OH)_2_D_3_ can regulate glial cell line-derived neurotrophic factor (32) and nerve growth factor (33) expression. The ability of 1,25(OH)_2_D_3_ to regulate certain neurotrophic factors and influence inflammation has led to the hypothesis that 1,25(OH)_2_D_3_ is neuroprotective (34). In fact, a previous study has shown a reduction in reactive oxygen species-induced cell death and increased anti-oxidant species in glial cells by 1,25(OH)_2_D_3_ (35). Vitamin D insufficiency is associated with other neurological disorders, including Parkinson disease, schizophrenia, depression and cognitive decline (36), suggesting its essential role in maintaining normal CNS function. The importance of vitamin D in CNS development is well-documented (37). While the anti-inflammatory roles of vitamin D have been established in EAE and MS, the effect of vitamin D insufficiency in the central nervous system during early life have not been studied. IL-34 is a cytokine essential for the survival and homeostasis of microglia, it is predominantly expressed by neurons in the CNS, and its expression is highest during post-natal development and declines in adults (38–40). IL-34 shares a common receptor, CSF-1R, with CSF-1, and all are robustly but differentially expressed in the CNS (41). We hypothesized that vitamin D signaling in neurons may promote IL-34 production that helps prevent excessive microglia activation during inflammation in early life, making the CNS less vulnerable to CNS autoimmunity.

## Method

### Cell Line Cultures

The mouse neuroblastoma (N2a) cell line was thawed and cultured for 7 days in Dubecco's Modified Eagle's Medium (DMEM) with 10% Fetal Bovine Serum (FBS). The cells were then treated with 10 nM retinoic acid for an additional 7 days to promote differentiation into neuronal-like cells. The cells were washed and treated with calcitriol at 0–1,000 nM. The neuron-conditioned media (NCM) was collected at various time points, and the cells were lysed in 1 mL of TRIzol and stored at −80°C to be used for real-time PCR.

The murine microglial (BV-2) cell line was thawed and cultured in DMEM with 10% FBS for 7 days. The cells were activated with LPS (10 ng/ml) and/or NCM from the N2a cell line as detailed in the figure legend. The supernatants were collected and stored at −20°C for ELISA analysis. The cells were lysed in 1 mL of Trizol at −80°C to be analyzed by real-time PCR.

### Primary Cell Cultures

Primary neurons were isolated from neonatal mice at post-natal day 1. The hippocampus was separated from the brain, and the meninges were removed. The tissue was minced, washed three times with PBS, and digested in trypsin (0.025%) for 15 min at 37°C. The cells were passed through a cell dissociation sieve and resuspended in neuronal medium which contained Neuronal Basal Medium with B-27 Serum-Free Supplement (2%), GlutaMAX-1 (0.5 mM), and gentamicin (20 μg/mL). The cells were plated at 200,000 cells per well in a 24 well-plate coated with poly-L-lysine and cultured for ~14 days with replacement of half of the medium every 3 days. Calcitriol (Sigma-Aldrich; D1530) was added to the neurons at a concentration of 0–1,000 nM on day 14. The cell lysate was collected for real-time PCR and supernatant was collected for ELISA at specified times.

Primary microglia were isolated from neonatal mice as previously described (42). In brief, neonatal brains were minced, digested in trypsin (0.025%) for 15 min at 37°C, and resuspended in 15 mL of microglial medium which contained DMEM with FBS (20%), gentamicin (20 μg/mL), L-glutamine (1%), and penicillin streptomycin (1%). The cells passed through a cell dissociation sieve and plated in a poly-L-lysine coated flask. The medium was replaced every 3 days and cultured for a total of 10 days at 37°C. These mixed glial cultures were then shaken for 3 h at 150 rpm at 37°C to recover the microglia. The microglia were plated on poly-L-lysine coated plates. After 48 h, the cells were then treated (1) for 24 h with NCM from primary neurons that had been treated with calcitriol, washed and activated with lipopolysaccharide (LPS, 0 or 10 ng/mL), or (2) with IL-34 (0, 2, or 10 ng/mL) and activated with lipopolysaccharide (LPS, 0 or 10 ng/mL). A half hour before supernatant collection, adenosine triphosphate (ATP) was added to the culture to release produced IL-1β. The supernatant was collected for ELISA analysis and stored at −20°C, and the cells were lysed in Trizol for real-time PCR analysis.

### Enzyme-Linked Immunosorbent Assay (ELISA)

Cell culture supernatants were analyzed for cytokines. Sandwich ELISA was performed as previously described (43, 44) with the following antibodies: IL-6 (capture: MP5-20F3; detection: MP5-32C11; BD Pharmingen), TNFα (capture: 6B8; detection: MP6-XT22; BioLegend), IL-1β (capture: B122; detection: Poly3138; BioLegend); IL-10 (capture: JES5-2A5; detection: SXC-1; BD Pharmingen), and IL-34 (capture: CO54-35; detection: Poly5193; BioLegend). In brief, ELISA plates were coated with a purified capture antibody, blocked, and incubated with culture supernatants overnight at 4°C. The plates were washed, incubated with a biotinylated detection antibody, and cytokines were detected using avidin-peroxidase and TMB Chromogen Solution (Life Technologies). Emax Precision Microplate Reader (Molecular Devices) was used to measure the enzymatic reaction in the ELISA and SoftMax Pro Software version 5.4.5 was used for analysis.

### Quantitative Real-Time PCR

Cells were lysed and stored in TRIzol (Ambion) at −80°C until analysis. RNA was isolated according to the manufacturer's instructions. RNA (2.5 μg) was reverse transcribed into cDNA using the SuperScript II Reverse Transcriptase according to the manufacturer's protocol. Each reaction contained 100 ng/μl random primer (1 μl), 10 mM dNTP (1 μl), 5X First-Strand Buffer (4 μl), 0.1 M DDT (1 μl), RNaseOUTTM (1 μl), and SuperScript II RT (1 μl). The mRNA levels were determined by real-time PCR using the Power SYBR Green PCR Master Mix (Applied Biosystems) according to the manufacturer's protocol. The results were normalized to 1 using the HPRT levels of the no treatment group. The reactions were initiated and read with the Applied Biosystems ViiA 7 Real-Time PCR System. The results were calculated using 2^−ΔΔCt^ method.

### Mouse Model

Swiss VDR^flox^ mice (45) were crossed with C57Bl/6 Single-neuron Labeling with Inducible Cre-mediated Knockout (SLICK-H; Jackson Labs) mice which delete VDR when administered tamoxifen in Thy1-expressing neurons and express YFP in Cre-expressing neurons (46). The SLICK transgene is designed with a modified *Thy1* promoter sequence that is required for neuronal expression, but lacking the sequence needed for non-neuronal cell expression, allowing for neuron-specific gene targeting. The SLICK mice were backcrossed 3 times onto the Swiss VDR^f/+^ background and EAE was induced in the F3 mixed-background mice. The mice were fed tamoxifen chow continuously from 3 to 5 weeks and then returned to standard chow. At 8–10 weeks, the mice were immunized s.c. with 50 μg MOG35-55 and 50 μg PLP139-151 homogenized in CFA containing 2 mg/ml *Mycobacterium tuberculosis*, followed by an i.p. injection with pertussis toxin (100 ng/mouse), or immunized s.c. with 12 μg MOG35-55 and 12 μg PLP139-151 homogenized in CFA containing 1 mg/ml *M. tuberculosis*, followed by an i.p. injection with pertussis toxin (25 ng/mouse). The mice were monitored for clinical signs of EAE according to the following scale: 0—no signs of disease; 1—flaccid tail; 2—moderate hind limb weakness; 3—severe hind weakness; 4—complete paralysis of at least one hind limb; 5—forelimb weakness or moribund; 6—death due to EAE (47).

### Immunohistochemistry

Cell culture: N2A, primary neuron and microglia were seeded on poly-L-lysine-coated cover glass in 24-well-plates. At the end of culture, the cells were fixed with 4% paraformaldehyde for 20 min at room temperature. After washing 3 times with PBS, the cells were permeabilized and blocked using 5% normal donkey serum + 0.3% Triton-X in PBS for 10 min at room temperature. The cells were then incubated with primary antibody diluted in 5% normal donkey serum + 1%BSA in PBST (PBS + 0.1% Tween 20) for 1 h at room temperature. N2A and primary neuronal cells were stained for β-tubulin (R&D Systems; TuJ-1), and microglia were stained for rabbit anti-Iba-1 (Wako Chemicals USA). After 3 washes, the cells were incubated with secondary antibody diluted in 1%BSA in PBST for 1 h at room temperature. After 3 washes, the cells were incubate on 0.05 μg/ml DAPI for 1 min and then rinsed with PBS. *Ex vivo*: The mice were perfused using 4% PFA with pH 9.4 and the brain and spinal cord was removed. The tissue samples were incubated in 10, 20, and then 30% sucrose each for 24 h. The tissues were frozen into Optimal Cutting Temperature (O.C.T) Compound and cut into 10 μm slices. The slides were stored at −20°C. The tissues were incubated in 0.4% Triton-X with PBS for 30 min to permeablize the sample. The tissues were then incubated in 1% BSA, 0.4% Triton-X, 5% goat serum, and 0.001% sodium azide in PBS from 1 h as a blocking solution. The primary antibody (Santa Cruz D-6, sc-13133) for vitamin D-receptor (VDR) was then added at a concentration of 1:500 in blocking solution and was incubated overnight. The slides were then washed three times for 10 min each in PBS. The secondary antibody, goat anti-rabbit was added at a concentration of 1:4,000 in blocking solution for 1 h. The slides were again washed three times for 10 min each. The slides were fixed with VECTASHIELD Hard Set Mounting Medium with DAPI. Pictures were taken at 40x with Olympus 8X41 microscope. ImageJ was used to identify VDR+ cells in wildtype mice, set the threshold for normal VDR expression, and identify the cells with significantly reduced VDR expression.

### Statistical Analysis

GraphPad Prism software was used to perform the statistical analysis. The unpaired *t*-test was used for all single comparisons and one-way ANOVA with Bonferroni's *post-hoc* analysis was used for multiple comparisons for *in vitro* studies. Significant changes in EAE clinical course was evaluated using the Mann-Whitney test.

## Results

**Figure 1:**
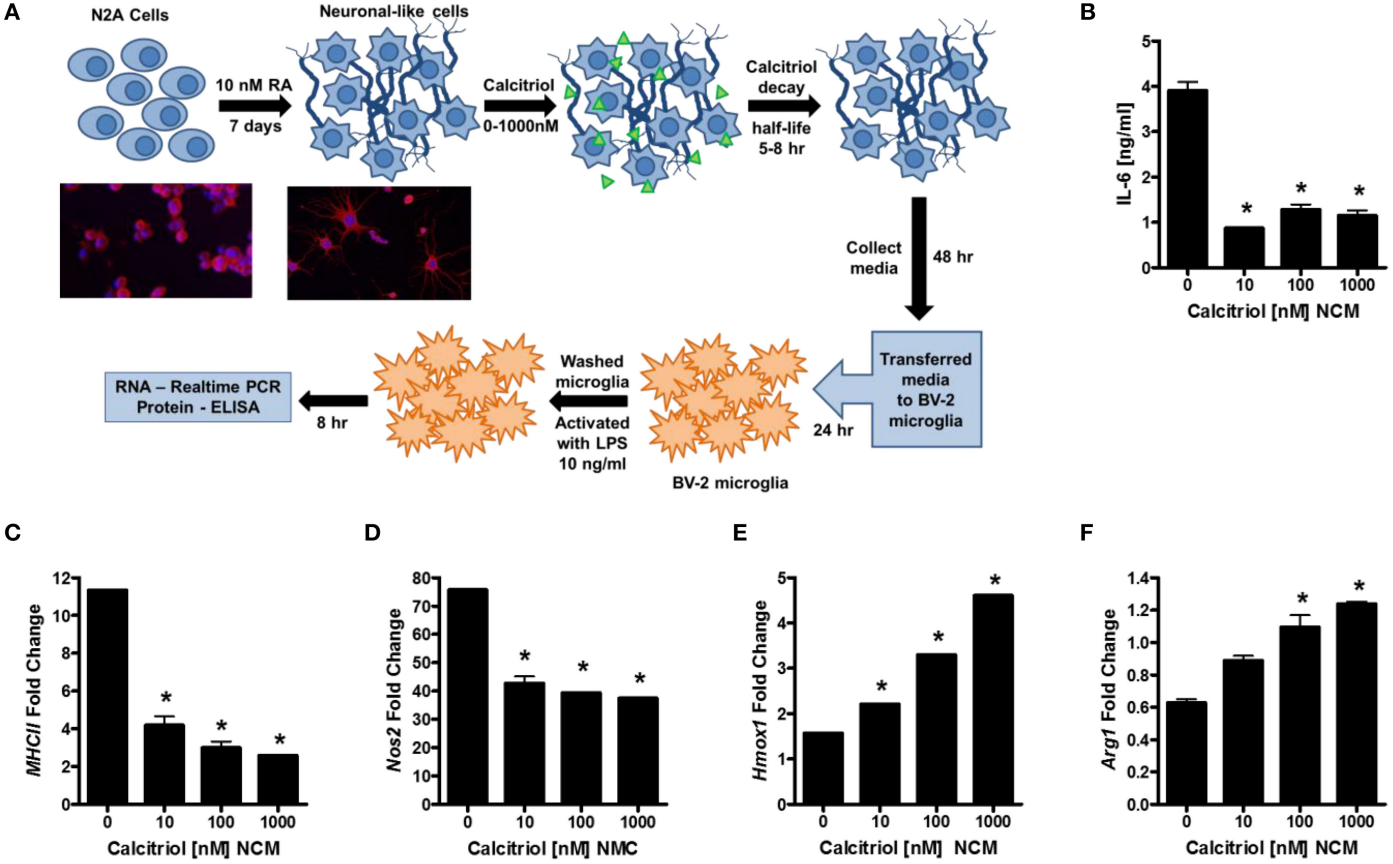
Vitamin D signaling in neurons reduces microglial activation. **(A)** N2a cells were differentiated into neuronal-like cells using retinoic acid, treated with calcitriol (0–1,000 nM), and the media collected (NCM). Micrographs illustrate the N2a cells before and after 7 days with retinoic acid stained for tuj1 [neuron-specific class III beta-tubulin (Red—β-tubulin; Blue—DAPI)]. The BV-2 microglia cell line was placed in culture, treated with NCM for 24 h, washed, and activated with LPS. After 8 h, IL-6 was measured in the BV-2 supernatant **(B)**, and transcripts for *MCHII* **(C)**, *Nos2* **(D)**, *Hmox1* **(E)**, and *Arg1* **(F)** were measured by real-time PCR in the microglia. *p < 0.05.

Our first question was whether vitamin D induces anti-inflammatory molecules in neurons. To this end, we differentiated murine N2a cells into neuronal-like cells with retinoic acid (RA; Figure 1A), treated the cells with calcitriol (the active form of vitamin D_3_), collected the supernatants, and evaluated the ability of the neuronal-conditioned media (NCM) to suppress inflammatory markers on the murine microglial cell line, BV-2. Calcitriol is relatively unstable with half-life only 5–8 h, and has been shown to be near depletion in culture after 2 days (48). BV-2 microglia were cultured with NCM from calcitriol-treated neurons and then activated with LPS. IL-6 was significantly reduced in LPS-activated microglia (Figure 1B), as well as *MhcII* and *Nos2* mRNA (Figures 1C,D), molecules associated with pro-inflammatory microglia. In contrast, transcript levels of anti-inflammatory molecules, Hmox1 and Arg1, were increased (Figures 1E,F), suggesting that calcitriol was inducing molecules in neurons that could reduce the pro-inflammatory phenotype and promote anti-inflammatory molecules in activated microglia.

**Figure 2:**
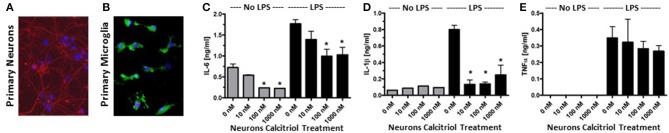
Vitamin D signaling in primary neurons reduces pro-inflammatory cytokine production by microglia. **(A)** Primary neurons were isolated from the hippocampus of post-natal day 1 mice. Red—β-tubulin; Blue—DAPI. **(B)** Primary microglia stained with Iba1 (green) and DAPI (blue). The primary neurons were cultured with calcitriol and the media was collected and transferred to the primary microglia. After 24 h, the microglia were washed, activated with LPS, supernatants collected, and IL-6 **(C)**, IL-1β **(D)**, and TNFα **(E)** were measured in the supernatants by ELISA. *p < 0.05.

To confirm that vitamin D induced anti-inflammatory molecules in neurons, cortical, and hippocampal neurons were isolated from P1 mice and cultured with calcitriol (Figure 2A). The NCM from the calcitriol-treated cortical neurons was transferred to the primary microglia (Figure 2B). After 24 h, the NCM was washed away and the primary microglia were active with LPS, resulting in a significant decrease in IL-6 and IL-1β (Figures 2C,D), but no effect on TNFα levels (Figure 2E). This confirmed that vitamin D induced anti-inflammatory molecules in primary neurons.

**Figure 3:**
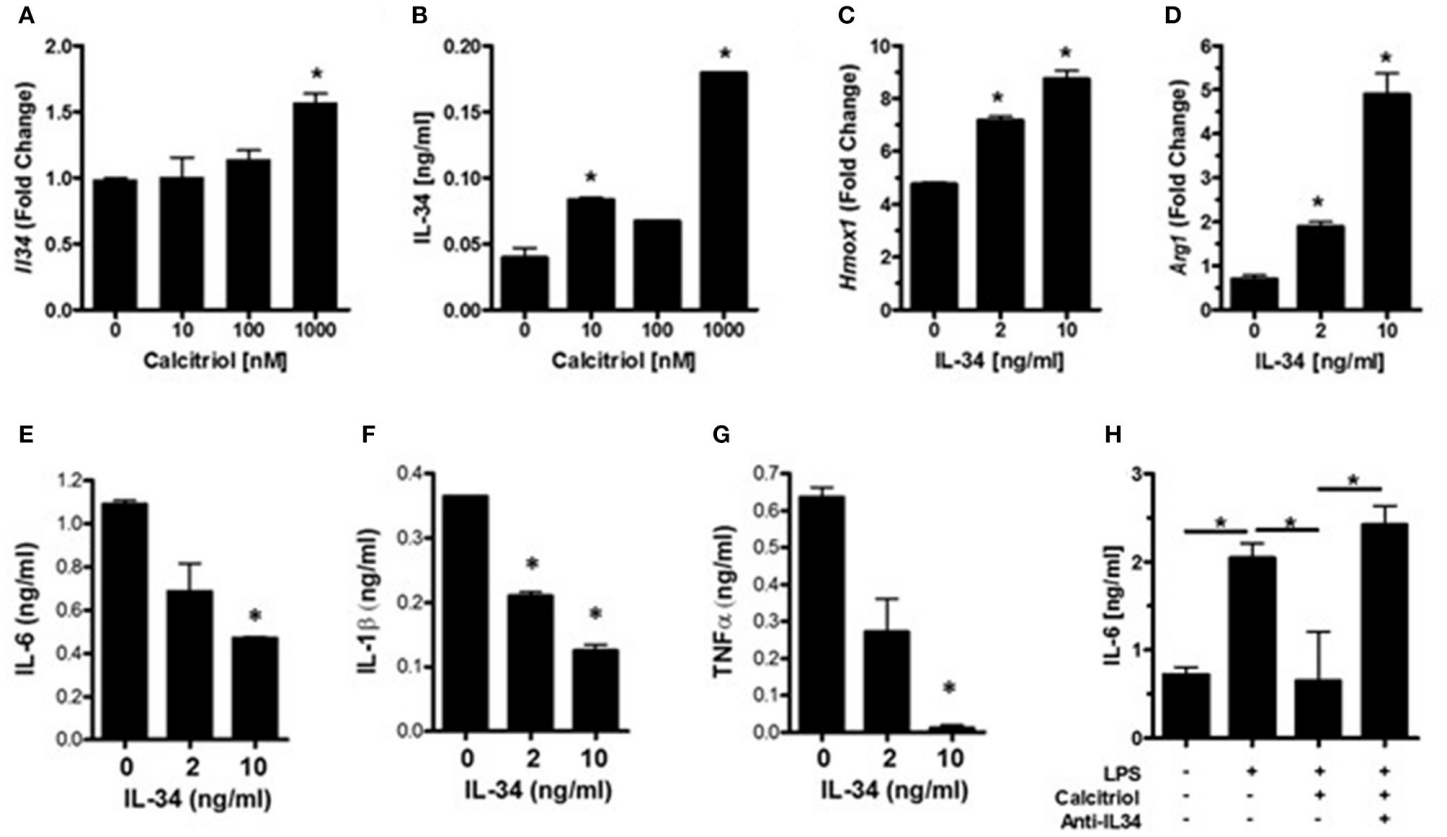
Vitamin D induces IL-34 in neurons, and IL-34 induces an anti-inflammatory phenotype in microglia. **(A)** IL34 transcripts were measured by real-time PCR in primary neurons treated with calcitriol. **(B)** IL-34 was measured by ELISA in the supernatants of primary neurons treated with calcitriol. *Hmox1* **(C)** and *Arg1* **(D)** transcripts were measured by real-time PCR in primary microglia treated with IL-34 and activated with LPS. IL-6 **(E)**, IL-1β **(F)**, and TNFα **(G)** were measured in the supernatants of primary microglia treated with IL-34 and activated with LPS. **(H)** NCM from calcitriol-treated primary neurons was treated with anti-IL34, transferred to primary microglia, and IL-6 was measured by ELISA. *p < 0.05.

IL-34 is a survival factor for microglia and was found to be induced by vitamin D in endothelial cells (49). Since neurons are the major source for IL-34 in the CNS (38), we hypothesized that vitamin D may induce IL-34 production in neurons and that IL-34 may be important for minimizing microglial activation during an insult. Analysis of IL-34 transcript levels in calcitriol-treated primary neurons found that there was a dose-dependent increase in IL-34 (Figure 3A), although only high concentrations of calcitriol resulted in a significant increase in *Il34* mRNA levels. Calcitriol induced IL-34 expression, but the amount was relatively low (Figure 3B). To determine if IL-34 induces anti-inflammatory properties in microglia, primary microglia were isolated, and stimulated with LPS in the presence of IL-34. *Hmox1* and *Arg1* mRNA levels were significantly increased (Figures 3C,D), indicative of an anti-inflammatory phenotype. The amount of IL-6, IL-1β, and TNFα were all reduced, suggesting that IL-34 was suppressing pro-inflammatory cytokine production (Figures 3E–G). A recent study found that calcitriol could induce IL-34 in a human neuroblastoma cell line (SH-SY5Y), and identified a putative VDRE site in the IL-34 promoter (50), supporting the hypothesis that vitamin D can positively regulate IL-34 expression.

To determine if IL-34 was potentially responsible for the anti-inflammatory phenotype induced by the calcitriol NCM observed in Figure 2, a neutralizing antibody to IL-34 was added to the NCM prior to the NCM being added to primary microglia. Blocking IL-34 reversed the effects of the calcitriol NCM on IL-6 production (Figure 3H). However, neutralizing IL-34 with anti-IL-34 had little, if any, effect on most of the inflammatory markers, suggesting that IL-34 may contribute to the suppression of IL-6, but there are other calcitriol-induced molecules that contribute to a broad anti-inflammatory phenotype.

**Figure 4:**
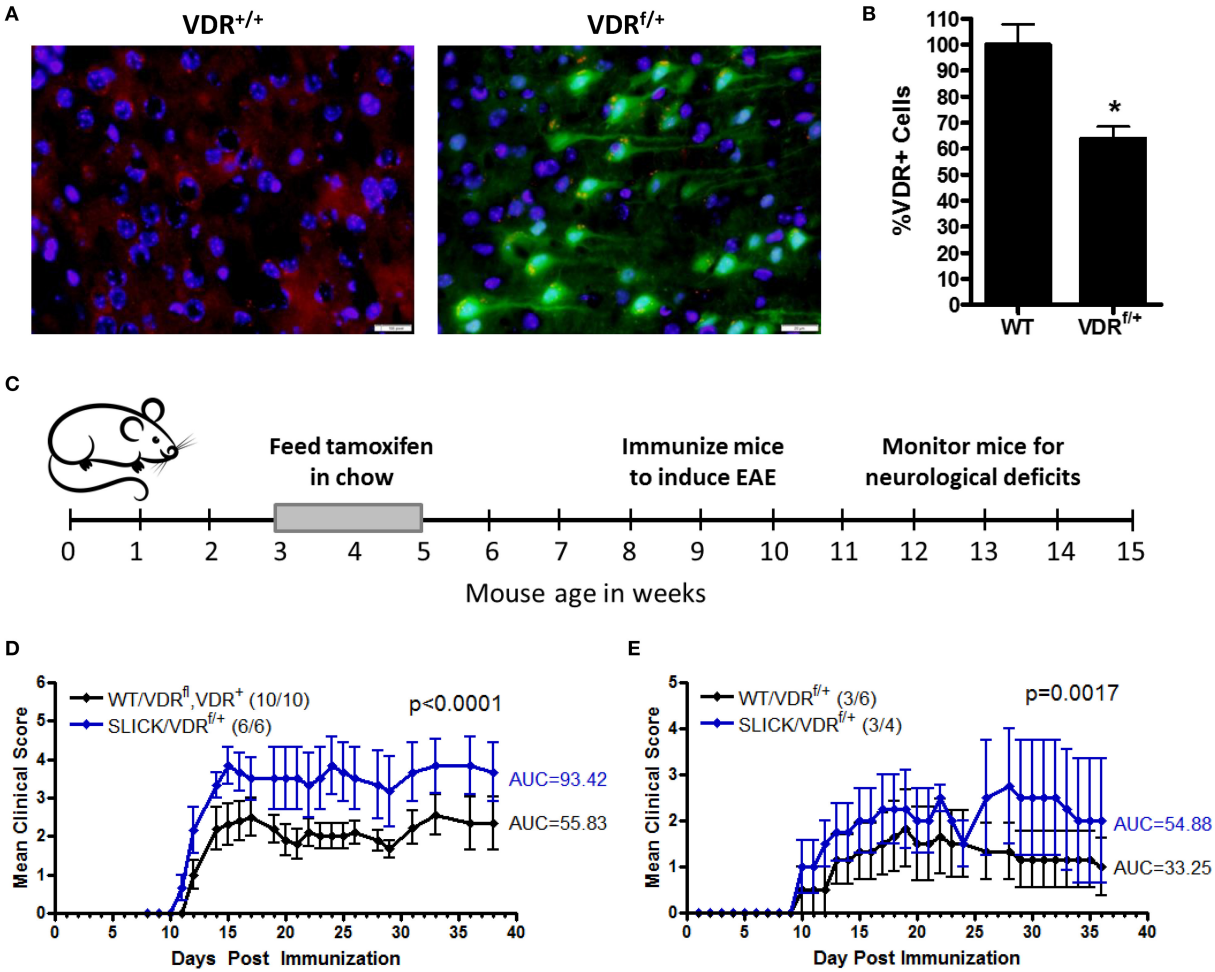
Reduced VDR signaling in neurons during early life enhances CNS autoimmunity. VDR^f/+^ mice were crossed with SLICK-H [Tg(Thy1-cre/ERT2,-EYFP)HGfng/PyngJ] to generate SLICK/VDR^f/+^ mice. Experiments used littermates and all mice were fed tamoxifen chow from 3 to 5 weeks of age and then returned to standard chow. **(A)** The brains and spinal cords were analyzed for VDR expression—red = VDR, green = Cre-EYFP, blue = DAPI. **(B)** Quantification of VDR+ cells in the brain and spinal cord, normalized to WT mice. *N* = 7 per group, *p < 0.05. **(C,D)** Mice were fed tamoxifen chow at 3–5 weeks and immunized at 8–10 weeks with 50 μg MOG35-55 and 50 μg PLP139-151 homogenized in CFA containing 2 mg/ml *Mycobacterium tuberculosis*, followed by an i.p. injection with pertussis toxin (100 ng/mouse). Controls = 10 and SLICK/VDR^f/+^ = 6 mice. *p* < 0.0001 for EAE scores (Mann-Whitney). **(E)** Mice were fed tamoxifen chow at 3–5 weeks and immunized at 8–10 weeks with 12 μg MOG35-55 and 12 μg PLP139-151 homogenized in CFA containing 1 mg/ml *Mycobacterium tuberculosis*, followed by an i.p. injection with pertussis toxin (25 ng/mouse). Controls = 6 and SLICK/VDR^f/+^ = 4 mice. *p* < 0.0017 for EAE scores (Mann-Whitney). Data is representative of ≥3 experiments.

Since low vitamin D during childhood is a risk factor for MS, we investigated the contribution of vitamin D insufficiency in early life in a model of MS. To distinguish the effects of vitamin D on immune cells and neurons, and to control the timing and duration of vitamin D insufficiency, we generated an inducible neuron-specific deletion model by using a tamoxifen-inducible vitamin D receptor (VDR)-flox system, VDR^f/+^ mice were crossed with SLICK-H [Tg(Thy1-cre/ERT2,-EYFP)HGfng/PyngJ] to generate SLICK/VDR^f/+^ mice which should have reduced VDR signaling in neurons when treated with tamoxifen, but not a total loss of VDR signaling, comparable to what would be observed in humans. To mimic vitamin D-insufficiency in children, the mice were fed tamoxifen at 3–5 weeks of age and switched back to normal chow. Reduction of VDR expression was verified in the CNS in tamoxifen-fed SLICK/VDR^f/+^ mice (Figure 4A). There was a ~35% reduction in the number of cells with normal VDR expression (Figure 4B). To determine if the SLICK/VDR^f/+^ mice in which VDR had been partially deleted in early life (3–5 weeks old) had an altered susceptibility to CNS autoimmunity, experimental autoimmune encephalomyelitis (EAE) was induced at 8–10 weeks of age using a suboptimal EAE induction protocol in which the amount of peptide (100 μg), mycobacterium concentration (2 mg/ml) in CFA, and pertussis toxin (100 ng) were used at 50% of our standard protocol, allowing for a change in disease incidence or severity to be more readily observed (Figure 4C). The disease severity was significantly enhanced in the SLICK/VDR^f/+^ mice compared to the littermate control mice which included mice that were WT for cre and had the VDR-flox or VDR-WT allele, all of which were fed tamoxifen (Figure 4D). Since 100% of mice still developed EAE with this protocol, we further reduced the peptide (24 μg), mycobacterium concentration (1 mg/ml), and pertussis toxin (25 ng) to determine if low neuron-specific VDR signaling could alter disease incidence. Using this protocol, SLICK/VDR^f/+^ mice had a higher incidence of EAE (75%) compared to WT/VDR^f/+^ mice (50%), as well as an increase in disease severity (Figure 4E). The exacerbated EAE in the SLICK/VDR^f/+^ mice suggest that vitamin D signaling in neurons protects the CNS from inflammation and minimizes the risk of CNS autoimmunity.

## Discussion

The goal of this study was to determine if vitamin D signaling in neurons contributed to an anti-inflammatory environment that minimized microglia activation and reduced the risk of CNS autoimmunity. MS is a complex neuro-immune disease and role of vitamin D in the risk of MS has largely been contributed to the immuno-modulatory effects of vitamin D. However, vitamin D signaling is important for normal CNS development (36). Thus, it seemed important to determine if the increased risk of developing MS in adult individuals with vitamin D-insufficiency in childhood was potentially due to a role for vitamin D in the CNS. Our initial hypothesis was that vitamin D signaling in neurons was important for the expression of IL-34, a critical cytokine for the survival and homeostasis of microglia, and that reduced vitamin D signaling in neurons would promote an inflammatory environment that made the CNS vulnerable to autoimmunity. This hypothesis was based on several previous observations. First, vitamin D was found to induce IL-34 expression in endothelial cells (49), and IL-34 plays a role in restoring blood-brain barrier by enhancing the expression of tight junction proteins in endothelial cells (51). Given that neurons are the primary source of IL-34 in the CNS, it seemed logical that vitamin D would be potentially important in the induction of IL-34 in neurons. Second, many common childhood viruses are neurotrophic, yet cause no clinical neurological manifestations, suggesting that the CNS of children is resilient to these subclinical CNS infections, perhaps due to mechanisms to minimize CNS inflammation. However, when vitamin D is low in children, the CNS may have enhanced inflammation and damage that may make the CNS more vulnerable to autoimmunity.

While vitamin D signaling in neurons did enhance IL-34 expression, it was modest. Furthermore, neutralizing IL-34 in calcitriol NCM only significantly reversed the changes in IL-6 levels, while most other pro-inflammatory and anti-inflammatory molecules expressed by microglia were not significantly altered by anti-IL-34. Thus, vitamin D signaling appears to reduce inflammatory mediators and promote the expression of anti-inflammatory molecules, but IL-34 is only a minor contributor to these vitamin D-induced changes. However, direct addition of IL-34 to primary microglia indicates that IL-34 does have the capacity to reduce inflammatory mediators and promote the expression of anti-inflammatory markers. Furthermore, the fact that NCM from neurons cultured with calcitriol could significantly change the phenotype of LPS-activated microglia indicates that neuron-specific vitamin D signaling plays an active role in controlling CNS inflammation.

To more directly address the question as to whether neuron-specific vitamin D signaling in early life is playing a role in the risk of developing CNS autoimmunity, we utilized mice that have an inducible and conditional reduction in VDR in neurons. This allowed for normal development of the CNS during embryogenesis and the neonatal period. Tamoxifen was administered via the chow at 3–5 weeks, a time comparable to 6–14 years in humans. Reduction in VDR in neurons during early life made mice more susceptible to EAE, indicating that VDR signaling in neurons is somewhat protective to CNS autoimmunity. A previous study found that feeding juvenile rats vitamin D-enriched diets protected them from EAE, while there was no benefit to adult mice (30).

This study is the initial step in understanding the role of vitamin D in protecting the CNS from inflammation and the risk of developing CNS autoimmunity. There are several limitations of this pilot study. First, despite the short half-life of calcitriol, there could be residual calcitriol in the NCM that could be have a direct effect on microglia. Second, the molecule(s) that are induced by calcitriol in neurons that mediate the anti-inflammatory effects have yet to be elucidated, other than the modest effect of IL-34. It is also unclear whether calcitriol is directly or indirectly inducing the anti-inflammatory mediators. There are still numerous questions to address. What are the neuroprotective molecules induced by VDR signaling in neurons? Does loss of VDR in neurons in adult mice affect EAE incidence or severity? Does reduced VDR signaling in other CNS cell types alter susceptibility to CNS autoimmunity? Understanding the role that vitamin D plays in CNS development, maturation, inflammation, neuroprotection, and repair is critical to understanding the pathology of MS and other neurodegenerative diseases.

## Data Availability Statement

The raw data supporting the conclusions of this article will be made available by the authors, without undue reservation, to any qualified researcher.

## Ethics Statement

This animal study was reviewed and approved by the Ohio State University Institutional Animal Care and Use Committee.

## Author Contributions

PL, AS, and SL designed and conducted experiments, analyzed data, and help prepare the manuscript. YL and YY provided technical expertise and edited the manuscript. AL-R designed the experiments, analyzed data, and wrote the manuscript.

### Conflict of Interest

The authors declare that the research was conducted in the absence of any commercial or financial relationships that could be construed as a potential conflict of interest.
